# 

**Published:** 1894-01-27

**Authors:** 


					t&e hospital. January 27th, 189
WITH EXTRA NURSING SUPPLEMENT.
Per Annum, 8s. 6d., post free.
Monthly Farts, 6d.; post free, 8d.
Ditto per Annxun, 8s? post free.
L A SCO Yi V/ESTER N 1N riRMARy"'^~ ^^ !HlibFOLli aub litlRWICH HOSPUAL
x ]
No. 383. Vol. XV. WITH EXTRA NURSING SUPPLEMENT. Saturday,
January 27th, 1894.

				

